# Comparative study of pelvic sarcoma patients undergoing internal and external hemipelvectomy: A meta-analysis study

**DOI:** 10.3389/fsurg.2022.988331

**Published:** 2022-10-14

**Authors:** Nishant Banskota, Hongsheng Yang, Xiang Fang, Dechao Yuan, Wenli Zhang, Hong Duan

**Affiliations:** Department of Orthopedics, West China School of Medicine/West China Hospital, Sichuan University, Chengdu, China

**Keywords:** internal hemipelvectomy, external hemipelvectomy, five-year survival rate, meta-analysis, local recurrence

## Abstract

**Introduction:**

Malignant and giant pelvic tumors are complex and rare, and hemipelvectomies are complex procedures performed for this malignant lesion. Only a few studies had been conducted on the survival and recurrence of pelvic sarcomas patients undergoing internal or external hemipelvectomy. In the present study, we compared internal with external hemipelvectomy in pelvic sarcomas on clinical outcomes by a meta-analysis.

**Methods:**

The survival and recurrence rates of pelvic sarcomas patients were collected from research reports from CNKI, MEDLINE, EMBASE, the Cochrane Database, and Google Scholar until April 2022. The quality of included articles was evaluated by two independent reviewers. Differences between patients undergoing internal and external hemipelvectomy were analyzed based on postoperative survival and recurrence rates.

**Results:**

Five articles were included according to selection criteria. There were 183 patients in total from these studies. Our results showed that there was no significant difference between limb salvage surgery and amputation according to survival; however, patients with internal hemipelvectomy had a lower recurrence rate.

**Conclusions:**

Internal hemipelvectomy results in a lower recurrence rate and similar survival rate, while not increasing the risk of metastasis and complications. This study provided more pieces of evidence to support internal hemipelvectomy as a favorable treatment of pelvic sarcomas.

## Introduction

Hemipelvectomy is a major orthopedic surgical procedure indicated in specific situations and regularly performed in advanced tertiary centers (1). Hemipelvectomy is commonly performed for soft tissue and bone sarcomas of the pelvis region (2). The reconstruction after hemipelvectomy is of importance for the later outcome and quality of life (3). Previously treatment of these tumors has been difficult because of the poor prognosis and the necessity for amputation (4). Hemipelvectomy involves the following two different approaches: external approach (with limb amputation) and internal approach (with limb preservation) and further internal approaches are divided into four subtypes based on anatomical location (3).

In recent years, the use of external hemipelvectomy for the treatment of pelvic tumors has declined, and new surgical techniques and efforts for resection with limb preservation (internal hemipelvectomy) and reconstruction have been introduced (5, 6). This major development in the medical field demands comparisons between these two vastly different procedures, as both procedures have their advantages and disadvantages. Survival and complications after hemipelvectomy might be related to several different factors, such as tumor size and histopathology, disease stage, patient general condition, and resection type (7). In patients with pelvic tumors, the 5-year survival rate and recurrence are expected to be high in number. Large tumors and bone and vascular involvement might be indicators of poor survival (8). A large previous study reported a survival rate of 50% after hemipelvectomy (9). Reoccurrence and metastasis also mainly depend upon tumor stage and resection.

There are not many studies focusing on these procedures and analyzing their short comes and benefits. There is a need for a study elaborating on these because of the poor quality of life that patients suffer after this extensive surgery. We conducted a meta-analyses study on survival, local reoccurrence, and metastasis in patients with pelvic tumors undergoing internal and external hemipelvectomy. In addition, our study focused on whether patients undergoing internal hemipelvectomy had a better 5-year survival rate and less recurrence, metastasis, and complications than external hemipelvectomy.

Through searching more abundant hemipelvectomy literature, we conduct this meta-analysis to get a comprehensive conclusion in hemipelvectomy patients treated with external and internal approaches. These results will help us to establish the most appropriate method to treat a tumor in the pelvic region. In our study, internal hemipelvectomy was set as the experimental group and external hemipelvectomy as a control group.

## Methods

**Figure 1:**
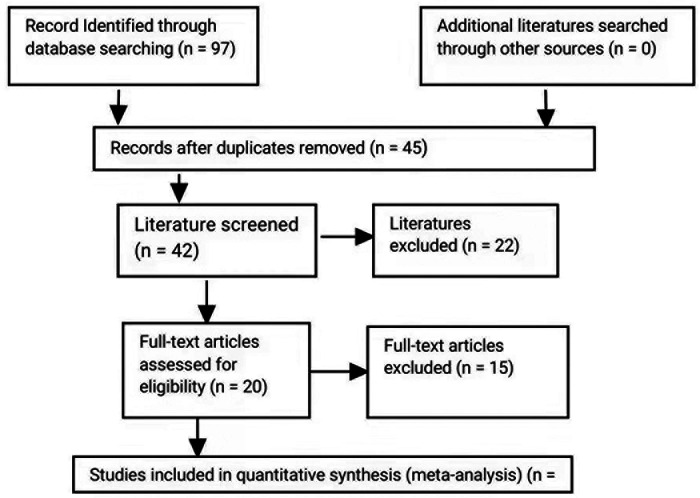
Flow chart of studies included and excluded.

This study was performed according to the Preferred Reporting Items for Systematic reviews and Meta-Analyses (PRISMA) guidelines (Figure 1) (10).

### Literature search

PUBMED, MEDLINE, Cochrane, EMBASE, and Google Scholar databases were searched for relevant data until April 30, 2022. The reference studies of relevant studies were also searched on different databases. Searches were expanded to 35 years, because of the lack of the study published on relevant topics. Keywords used for searching included internal hemipelvectomy, external hemipelvectomy, pelvic tumor, survival, recurrence, complications, and metastasis.

### Included studies

*Inclusion criteria:*

(1) English language studies including patients diagnosed with pelvic tumors;
(2) Use of internal and external hemipelvectomy for pelvic tumors; and
(3) Studies providing information on the 5-year survival rate, recurrence rate, metastasis, and complication after these two surgeries.

*Exclusion criteria:*

(1) Non-English studies;
(2) Non-comparative studies between internal and external hemipelvectomy;
(3) Case reports, review, letter to the editors; and
(4) Studies that lack adequate clinical data.

### Study selection and data extraction

Outcomes were collected from the articles by three authors of our study. The authors made a descriptive and informative table and then collected all the data into a database. The following data were extracted from articles according to the inclusion criteria: the name of the first author, year of publication, design scheme, number of patients in each group, patients' age and gender, and short and long-term after surgery. Data were extracted for (a) demographic characteristics, (b) 5-year survival rate, (c) recurrence rate (local and distant recurrence), (d) Metastases local and distant metastases), and (e) complications (wound complications, genitourinary complications, and flail hip).

### Quality assessment and outcome measurement

Literature focusing on similar research issues was included, and all studies were retrospective. In this study, the authors attempted to include randomized control trial (RCT) and prospective studies for a better outcome of the study, but the authors could not find any studies matching our criteria due to minimal studies published in this section. All studies had a low bias as studies were moreover similar with similar inclusion criteria, similar surgical procedures, and study periods. Inconsistencies were resolved on the assessment by the corresponding author. Quality assessment was done by the Newcastle-Ottawa Scale (NOS) (11) and the table is shown in the Supplementary File. In our study, the primary outcome was set as a 5-year survival rate and the secondary outcomes in our study were local recurrence, metastasis, and complications. The 5-year survival rate is defined operated patient having a life expansion of a minimum of 5 years after surgery.

### Statistical analysis

The outcome of measurement used in our study was the 5-year survival rate, local recurrence, metastasis, and complications which were all dichotomous data. We used the software of the Cochrane Collaboration (ReviewManager5.2) to calculate odds ratios (ORs) and 95% confidence intervals (CIs) for all outcomes. Statistical heterogeneity among the included studies was defined by the *I*^2^ tests. Statistically, significant heterogeneity was defined as an *I*^2^ value >0.5 (12). *I*^2^ illustrates the percentage of the total variability in effect estimates among trials that is because of heterogeneity rather than coincidence (13). Heterogeneity was defined as low, moderate, and high based on the *I* square value (<40%: low; 30%–60%: moderate; 50%–90%: substantial >75%: high). Heterogeneity with a high *I* square value >50% was considered statistically significant. A random-effects model was selected for heterogeneous data; otherwise, a fixed-effect model was selected. Publication bias was identified through funnel plots, which exhibited the intervention effect from the individual study against the respective standard error. An asymmetrical plot suggested there was no publication bias, and any asymmetry of the plot suggests the existence of publication bias.

## Results

### Study selection

In the primary study search, 97 relevant articles were retrieved and 45 were excluded based on the exclusion criteria (Figure 1). The abstracts of the remaining 42 were screened, and 22 were excluded based on the exclusion criteria. After all the reviews of the remaining 20 studies, 10 were excluded due to lacking outcome of (*n* = 10) and duplication in the study population with other articles (*n* = 5). In a word, a total of five articles were included in the meta-analysis. Characteristics of the studies are summarized in Table 1, and outcomes are summarized in Table 2.

**Table 1:** Characteristics of the included studies.

Studies	Study period	Patient number	Male/Female	Median age	Study design	Newcastle-Ottawa Scale (NOS)	Country
Griesser 2011	2002–2007	15	11/4	46.9	Retrospective	8	United States
Guder 2015	1999–2012	34	21/13	70.2	Retrospective	8	Germany
Guo 2011	1996–2005	60	37/23	45.5	Retrospective	8	United States
Ham 1997	1970–1995	21	14/7	43	Retrospective	8	Netherland
Huth 1988	1974–1986	53	31/22	40	Retrospective	8	United States

**Table 2:** Outcomes of the included studies.

Reference	Local recurrence (internal/external)	5-year survival (internal/external)	Metastatic (internal/external)	Complications (internal/external)		
				Wound infection	GU	Flail hip
Griesser 2011	1/15		3/15	1/15	1/15	
Guder 2015	3/34	29/34	5/34			
Guo 2011	25/60		16/60	12/30		5/60
Ham 1997	5/21	14/21	8/21	5/21	3/21	4/21
Huth 1988	4/33	17/33		3/33		

GU, Genitourinary.

### Five-year survival rate and tests for heterogeneity

Among all the eligible studies, three of the five studies reported a 5-year survival rate. Data were recorded as patients not surviving for 5 years and the result was moreover similar in both groups. In the analysis of the fixed model effects, the *I*^2^ score was 76%, thus random-effect model was conducted. There was no significant heterogeneity in the comparison of 5-year overall survival between the internal and external hemipelvectomy groups (OR = 1.15, 95% CI 0.07–18.18, *P* = 0.93).

### Recurrence rate

**Figure 2:**
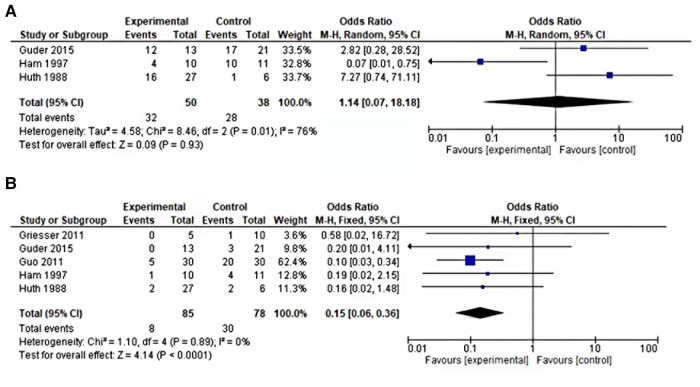
(**A**) Forest plot of comparison 5-year survival rate of internal vs. external hemipelvectomy in pelvic tumors. (**B**) Forest plot comparing local recurrence of internal vs. external hemipelvectomy in pelvic tumors.

All five studies reported recurrence. Recurrence occurred in all five studies either in the internal group or the external group. A fixed-effects model of analysis was used (14). There was a significant difference in the local recurrence rate between internal and external hemipelvectomy, fewer recurrences were seen in the internal group (OR = 0.15, 95% CI 0.06–0.36, *P* < 0.0001) as shown in Figure 2. Recurrence in our study included both local and distant recurrence.

### Metastasis

**Figure 3:**
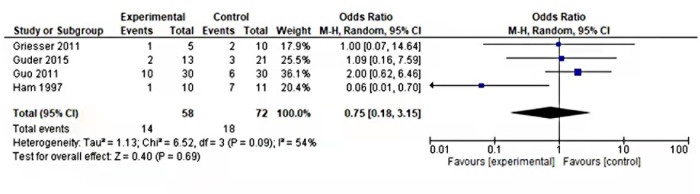
Forest plot of comparison metastasis of internal vs. external hemipelvectomy in pelvic tumors.

Among all the eligible studies, four of the five studies reported metastasis. In our studies, both distant and local metastases were included in metastases titled outcome. The outcome was moreover similar in both groups suggesting no significance relating to this outcome (OR = 0.89, 95% CI 0.40–1.96, *P* = 0.77) as shown in Figure 3. .

### Complications

**Figure 4:**
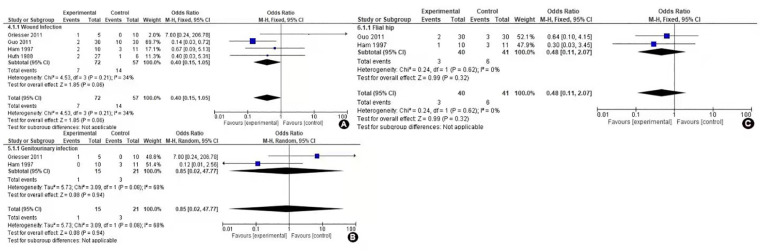
Forest plot of comparison complications of internal vs. external hemipelvectomy in pelvic tumors: (**A**) Wound complication, (**B**) genitourinary complications, and (**C**) flail hip.

Many local and systemic complications are associated with both these procedures; our studies only included three complications wound, genitourinary, and flail hip which were moreover common in all our studies. Wound complications were reported in four of our included studies, more complications were associated with the external group than the internal group (OR = 0.40, 95% CI 0.15–1.05, *P* = 0.06). Genitourinary complications were also reported in four of our studies but were only recorded in two studies. The *I*^2^ value was recorded as 68%, hence analysis was conducted through random effects. The outcome was moreover similar in both groups (OR = 0.85, 95% CI 0.02–47.77, *P* = 0.08). Flail hip was also reported in four of our studies but only recorded in two studies. These complications less occurred in the internal group than external as suggested by the Forrest plot curve in Figure 4 (OR = 0.48, 95% CI 0.11–2.07, *P* = 0.32). As suggested by the *P*-value, there were not any significant results, but still, there were few complications associated with internal hemipelvectomy thus favoring the experimental group.

### Sensitivity analysis

Sensitivity analyses indicated that included studies were performed to determine the reliability of the results, with each study removed in turn (15). The magnitude and dynamics of the combined estimates did not have any difference markedly with the exclusion of individual studies, indicating that the findings of the meta-analysis are reliable and the result obtained by conducting a meta-analysis is stable. The statistical value when the first study was excluded (OR = 0.71, 95% CI 0.01–70.38, *P* = 0.88), when the second study was only excluded (OR = 4.56, 95% CI 0.9–23.14, *P* = 0.07), and when the third study was only excluded (OR = 0.44, 95% CI 0.01–17.33, *P* = 0.66). All sensitivity analysis figures are shown in the Supplementary File.

### Publication bias

**Figure 5:**
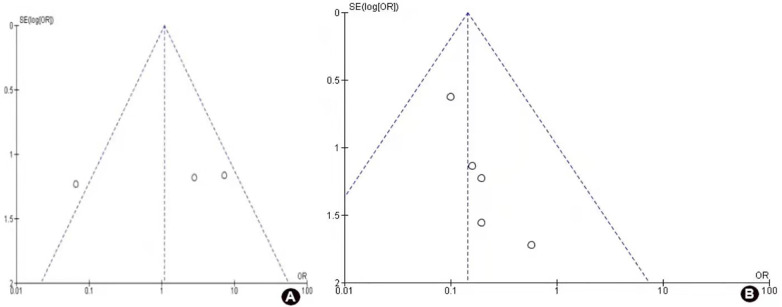
Funnel plot for publication bias, 4A. Five year survival rate, 4B. Local recurrence.

Funnel plots of the local recurrence rates and 5-year survival rates were shown in Figure 5. Funnel plots were used only in two primary outcomes of our studies which were local recurrence and 5-year survival rates. The findings showed that there is no evidence of publication bias for each of the two outcomes.

## Discussion

Malignant and giant pelvic tumors are aggressive and difficult to resect with unfavorable outcomes. The anatomical location makes it more complex and close and an adhered to major visceral organ adds to its poor prognosis (16). Most pelvic tumors are diagnosed at a late stage which also adds to their poor prognosis (17). Limb salvage surgery for malignant tumors of the pelvis is a formidable surgical undertaking, both from the viewpoint of surgical resection and reconstruction (18). The surgeon's primary goal is local control of the tumor by complete resection and the secondary goal is to preserve a functional limb (18). Many metastatic and malignant tumors can be observed in the pelvic region, due to their aggressive nature or extension to adjacent structures prognosis has been poor. Whether internal or external, hemipelvectomies are a major operative procedure and may be associated with significant functional impairments and morbidity including injury to the genitourinary tract, neurovascular injury, considerable soft tissue defects, blood loss, wound infections, and delayed wound healing (14, 19). Radiotherapy and chemotherapy have helped in improving the outcome of these major procedures. Adjuvant and neoadjuvant therapy are accepted treatments in the tumors of the pelvis region and the study conducted by Ng et al., justified this therapy by increasing the survival rate in Ewing sarcoma patients (20).

Any surgeon desires and aims to give hemipelvectomies patients a functional and comfortable postoperative life. There are very few studies comparing these procedures, as pelvic tumors are rare and many patients do not choose surgery as their treatment option due to its postoperative and financial burden. Chondrosarcoma is the most frequent primary tumor of the pelvis, followed by Ewing's sarcoma and osteosarcoma (21). Patients with these tumors seldom have desirable outcomes regardless of undergoing surgery or not. The survival rates in any tumor are often related to recurrence and metastasis, in the case of Ewing sarcoma 5-year survival is less than 10% (22). In a retrospective study by Shin et al., there was no significant difference between these two procedures based on survival and complications outcomes on a long-term basis, and found prognosis was better in lower-grade sarcomas (23). Survival is also influenced by older age (17) and associated comorbidity. A reconstructive procedure helps in maintaining joint stability but is associated with more complications (5, 24). Minimal studies have been conducted comparing the functional outcomes of these two procedures; a retrospective study by Guo et al., found that internal hemipelvectomy patients had better functional outcomes, shorter lengths of stay, and were early ambulators (2). Extensive muscle and soft tissue resection in external hemipelvectomy may have been an influencing factor in eliciting the results of this retrospective study (2). A retrospective study done by Apffelstaedt et al., reviewed 68 external hemipelvectomies and 32 internal hemipelvectomies and their study was focused on surgical complications and mobility after these procedures (25). Their total mortality rates from the surgery were 6% for external hemipelvectomies and 9% for internal hemipelvectomies (25). With respect to mobility, external hemipelvectomy patients as expected were in crutches with prosthesis or without prosthesis, and among that 9% of patients were wheelchair bound and 6% were bedridden (25). In another study conducted by Beck et al., quality of life was compared using the linear analog self-assessment (LASA) subcategory among these two procedures; no differences were noted between groups for any parameter except pain severity. Participants with external hemipelvectomies experienced a higher level of pain (26).

In our study only, three studies (4, 27, 28) reported 5-year survival rates and the outcome were moreover similar in both groups (OR = 1.15 *P*-value = 0.93). Then in the heterogeneity test, one large study (27) was excluded, there was apparent heterogeneity as findings were moreover similar. In contrast to our study, a retrospective study by Couto et al. found that the 5-year survival rate was significantly lower in patients who underwent external hemipelvectomy than in those who underwent internal hemipelvectomy (*P* = 0.043) (7). In the context of the internal approach comparative research are very few and hard to distinguish on an anatomical basis which internal approach has a better prognosis, a study done by Penna et al., suggested type I and III resection has good survival outcomes (29). Local recurrence in our study was found less in the internal group compared to the external group (OR = 0.15 *P* = 0.89). Local recurrence may be associated with larger tumor size and the absence of neoadjuvant chemotherapy (19). Metastasis was also similar to recurrence and among three complications wound complication was the most common in our meta-analysis literature, which also corresponds to other studies (30, 31). Internal hemipelvectomy presents an alternative procedure in the struggle against pelvic tumors and an adequate and tumor-free resection margin is of great value for the long-term oncological outcome (3). External hemipelvectomy is currently performed in specific situations of more advanced diseases such as failed neoadjuvant therapy, severe deep infection, sciatic nerve, and femoral vessel infiltration, local tumor recurrence, improvement of the resection margin, and as a life-saving or palliative procedure could explain the higher chances of survival in the internal hemipelvectomy group (7). Although we did not notice any significant statistical, based on less recurrence and other outcomes moreover similar in both groups, this study may suggest as internal hemipelvectomy is a favorable procedure.

A few limitations of this meta-analysis should be illustrated. First, the lack of detailed and verified data from original studies made it hard to adjust estimates by age, menopausal, lifestyle, smoking, race, and so on, while more accurate analysis needed this kind of adjusting. Second, there was no detailed data on our primary outcomes survival and no additional data to analyze the functional mobility of the patients. Third, there were only limited studies, so it is hard to get a statistically significant result.

Otherwise, our meta-analysis also has some beneficial points. First, a systematic review of the association of survival, recurrence, and metastasis in pelvic sarcomas patients with internal or external hemipelvectomy treatment was statistically more powerful than any single study. Second, all of the retrospective studies had a high quality and conformed to our inclusion criteria. Third, even though included studies were few and without statistically significant results, our study highlighted the importance of limb preservation leading to quality of life and encourages more literature on these rare topics.

## Data Availability

The original contributions presented in the study are included in the article/Supplementary Material, further inquiries can be directed to the corresponding authors.
