# Supplementary material for: Comparative study of pelvic sarcoma patients undergoing internal and external hemipelvectomy: A meta-analysis study
Source: Front Surg. 2022 Oct 14;9:988331. doi: 10.3389/fsurg.2022.988331 (PMC9614061; doi:10.3389/fsurg.2022.988331)
Supplement: Supplementary file 1 [file Table1.docx]

| **Supplementary Table 1: Newcastle– Ottawa Quality Assessment Scale for Cohort Studies** | | | | | | | | | | | | |
| --- | --- | --- | --- | --- | --- | --- | --- | --- | --- | --- | --- | --- |
| Authors | Represent-ativeness of the exposed cohort | Selection of the non exposed cohort | Ascertain-ment of exposure | Demonstra-tion that outcome not present at start | Compara-bility of cohort  (2 points) | | Assessment of Outcome | Follow-up length adequate for outcome to occur | Adequacy of Follow-up of cohorts (accounted for non-index hospitals) | Total Score  (9 points possible) | |  |
| Griesser 2011 | 1 | 1 | 1 | 1 | 1 | 1 | | 1 | 1 | | 8 |  |
| Guder 2015 | 1 | 1 | 1 | 1 | 1 | 1 | | 1 | 1 | | 8 |  |
| Guo 2011 | 1 | 1 | 1 | 1 | 1 | 1 | | 1 | 1 | | 8 |  |
| Ham 1997 | 1 | 1 | 1 | 1 | 1 | 1 | | 1 | 1 | | 8 |  |
| Huth 1988 | 1 | 1 | 1 | 1 | 1 | 1 | | 1 | 1 | | 8 |  |

Supplementary figure 1: Funnel plot for publication bias

1. Five-year survival rate


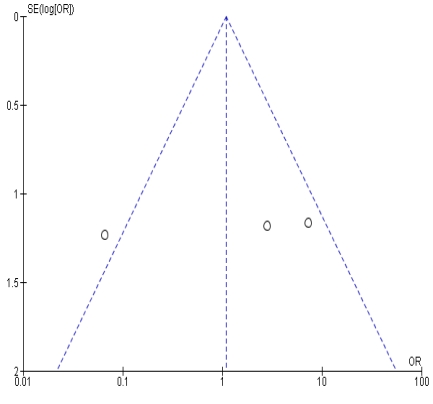


1. Recurrence rate


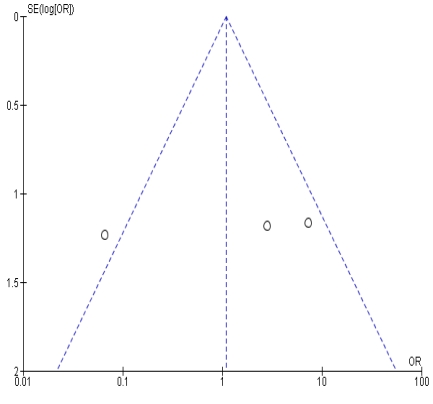


1. Metastases


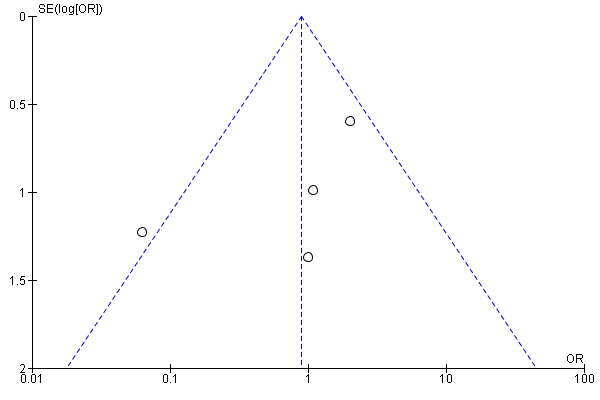


1. Wound infection


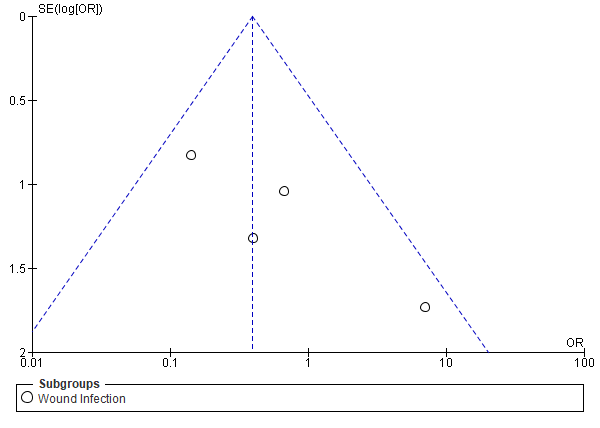


1. Genitourinary infection


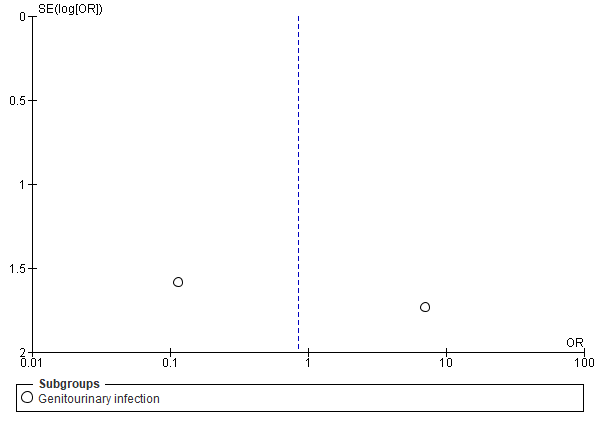


1. Flial hip


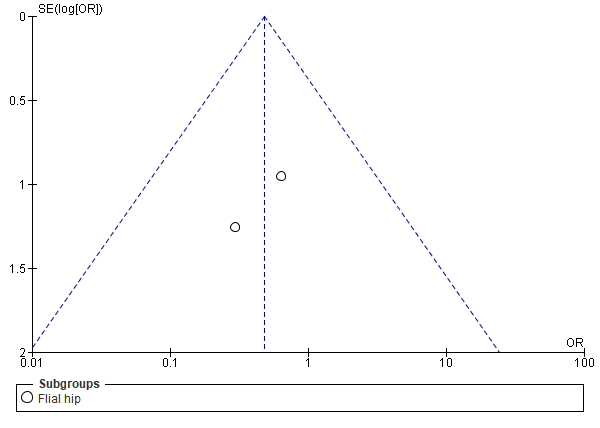


Supplementary figure 2: Results of leave-one-out sensitivity analysis


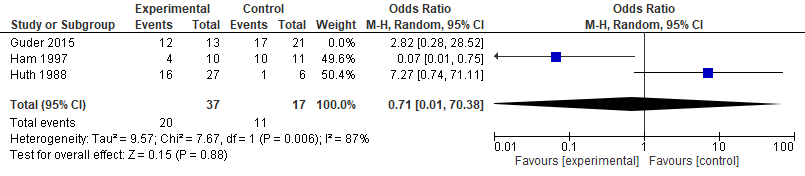


Supplementary Figure 2A: Forest plot for Five-year survival rate after excluding study by Guder et al.


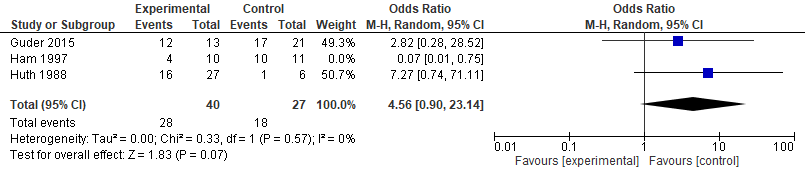


Supplementary Figure 2B: Forest plot for Five-year survival rate after excluding study by Ham et al.


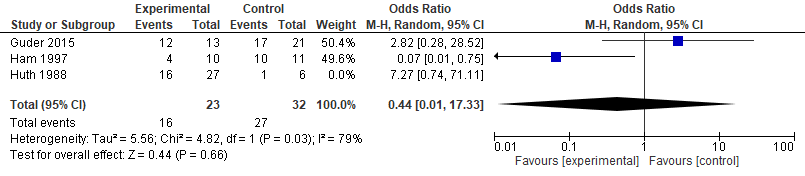


Supplementary Figure 2C: Forest plot for Five-year survival rate after excluding study by Huth et al.
